# AD|ARC: Construction of a research ready dataset to better understand farmers and farming households.

**DOI:** 10.23889/ijpds.v7i3.1905

**Published:** 2022-08-25

**Authors:** Jennifer Hampton, Nicholas Webster, Sophie Jordan, Sian Morrison-Rees, Orla Bateson, James Watson, Scott McFarlane, Alastair McAlpine, Liam Cavin

**Affiliations:** 1 Office for National Statistics; 2 ADR Wales, Welsh Government; 3 ADR Wales, Swansea University; 4 Northern Ireland Statistics and Research Agency; 5 Electronic Data Research and Innovation Service; 6 Rural and Environment Science and Analytical Services; 7 National Records Scotland

## Objectives

The AD|ARC Administrative Data: Agriculture Research Collection is an ambitious and original linkage project, bringing together information about farmers and farming households from several sources. When complete, this research-ready dataset will assist in addressing three broad themes: health and well-being, prosperity and resilience, and engagement with agri-environment.

## Approach

The dataset is being constructed from information drawn from survey, census, and administrative sources. Necessarily, this includes working across government departments to ensure comprehensive coverage of farm, business, education, and health data. Similarly, data owners, processors, and researchers are working closely to ensure the resultant dataset meets expectations. Alongside this cross-sectoral aspect, the work is also cross-jurisdictional, with the intention being for the data to capture information about farms, farmers and farming households from across the UK.

## Results

Rather than focus on the detail of the substantive research that AD|ARC will enable, this paper discusses some of the challenges and successes of this linkage project to date. Drawing on the experience of the teams from across the UK (England, Northern Ireland, Scotland, and Wales), the first part will discuss challenges faced in linkage of this multi-faceted project, alongside how the population census is being utilised to better understand farming communities, through the identification of both farming households and workers. Secondly, a broader discussion of the challenges and sensitivities of working across government departments and administrations will be presented, alongside ways of working developed to recognise and overcome these.

## Conclusion

The AD|ARC project will result in an invaluable resource to better understand the farming community, which in turn will help to better inform policy debate and decision making. Alongside this, the process of creating the dataset has offered opportunities for learning and insight across a range of issues.

